# Expression and Regulation of *PpEIN3b* during Fruit Ripening and Senescence via Integrating SA, Glucose, and ACC Signaling in Pear (*Pyrus pyrifolia* Nakai. Whangkeumbae)

**DOI:** 10.3390/genes10060476

**Published:** 2019-06-21

**Authors:** Haiyan Shi, Yuxing Zhang, Liang Chen

**Affiliations:** 1 Pear Engineering and Technology Research Center of Hebei, College of Horticulture, Agricultural University of Hebei, Baoding 071001, China, yyshhy@hebau.edu.cn; 2 Key Laboratory of Plant Germplasm Enhancement and Specialty Agriculture, Wuhan Botanical Garden, Chinese Academy of Sciences, Wuhan 430074, China

**Keywords:** pear (*Pyrus pyrifolia* Nakai. Whangkeumbae), ethylene insensitive3, salicylic acid, gene expression, fruit ripening and senescence

## Abstract

The economic value of fruit is reduced by having a short shelf life. Whangkeumbae is a type of sand pear (*Pyrus pyrifolia*) considered a climacteric fruit. The pear is famous for its smooth surface and good flavor. However, its shelf life is very short because of senescence and disease after harvest and a burst of ethylene (ET) production prompting the onset of fruit ripening. In plants, ETHYLENE INSENSITIVE3 (EIN3) and EIN3like (EIL), located in the nucleus, are important components of the ET signaling pathway and act as transcription factors. *EIN3*s and *EIL*s belong to a small family involved in regulating the expression of ethylene response factor gene (*ERF*), whose encoding protein is the final component in the ET signaling pathway. The mutation of these components will cause defects in the ethylene pathway. In this study, one gene encoding an EIN3 was cloned and identified from Whangkeumbae and designated *PpEIN3b*. The deduced PpEIN3b contained a conserved EIN3 domain, a bipartite nuclear localization signal profile (NLS_BP), and an N-6 adenine-specific DNA methylase signature (N6_MTASE). PpEIN3b belongs to the EIN3 super-family by phylogenetic analysis. Quantitative RT-PCR (qRT-PCR) analysis revealed that *PpEIN3b* was preferentially expressed in fruit. Additionally, its expression was developmentally regulated during fruit ripening and senescence. Furthermore, *PpEIN3b* transcripts were obviously repressed by salicylic acid (SA) and glucose treatment in pear fruit and in diseased fruit, while it was significantly induced by 1-aminocyclopropane-1-carboxylic acid (ACC) treatment. Taken together, our results reveal the expression and regulation profiles of *PpEIN3b* and suggest that *PpEIN3b* might integrate SA, glucose, and ACC signaling to regulate fruit ripening and senescence in pear, which would provide a candidate gene for this regulation to obtain fruit with a long shelf life and improved economic value.

## 1. Introduction

Post-harvest shelf life is a crucial factor influencing the economic value of fruits, as a short shelf life causes losses from fruit harvest to sale. According to whether there is a burst of ethylene (ET) production for the onset of ripening, fruits can be divided into two types: Climacteric and nonclimacteric [1,2]. Pear belongs to the climacteric type and has a typical respiratory climacteric at the onset of ripening. ET plays a critical role during pear ripening and senescence. There are four ecotypes of pear in the world, sand pear (*Pyrus pyrifolia*), white pear (*Pyrus bretschneideri*), Akiko pear (*Pyrus ussuriensis*), and European pear (*Pyrus communis*). Whangkeumbae is a sand pear and considered a climacteric type fruit that has a typical burst of ET production at the onset of ripening at about 10 days after natural harvest [3,4]. The fruit is famous for its high quality, including a smooth surface and good flavor. However, the pear senesces and succumbs to disease easily after harvest, leading to a short shelf life.

ET, an important plant hormone, regulates fruit ripening, petal senescence after fertilization, the triple response of germinating seedlings, root hair formation, leaf expansion, and stress responses [5]. The biological effects of ET on fruit ripening and senescence are mostly achieved via the ET signaling pathway. The ethylene receptors CONSTITUTIVE TRIPLE RESPONSE1 (CTR1), ETHYLENE INSENSITIVE2 (EIN2), ETHYLENE INSENSITIVE3 (EIN3), and ethylene response factor (ERF) are five main components in the ET signaling pathway [6]. EIN3s and EIN3-LIKE proteins (EILs) are classified as a small family involved in the regulatory cascade and function downstream of EIN2 in the ET signaling pathway [7]. The mutation of these components will cause defects in the ethylene pathway.

In plants, EIN3 and EIL are located in the nucleus and act as transcription factors. Transcriptional control conferred by EIN3 is pivotal [8]. Members of the EIN3/EIL family are able to regulate the expression of other transcription factors and thus are directly involved in regulating fruit ripening and quality [9,10]. Furthermore, *EIL*s and *ERF*s participate in the transcriptional regulation of ripening-related genes during the fruit ripening process [11]. As a senescence-associated gene, *EIN3* accelerates age-dependent leaf senescence by directly repressing miR164 transcription in Arabidopsis [12]. Salicylic acid (SA) and its derivative acetyl salicylic acid (ASA) have been reported to inhibit ET production in pear [13,14], suggesting a role of SA as an antagonist to ET action. Nowadays, SA is considered an important endogenous phytohormone that participates in delaying fruit ripening [15,16]. For fruit senescence alone, lower concentrations of SA delayed the senescence of Huang Kum pear fruit by regulating superoxide dismutase (SOD) and peroxidase (POD) activity [17]. Moreover, SA inhibits ET biosynthesis [14] by suppressing 1-aminocyclopropane-1-carboxylic acid (ACC) oxidase activity [18] and regulating the expression of ACC oxidase (ACO) genes [19]. SA also regulates the expression of ACC synthase (ACS) genes, which encode the rate-limiting enzyme in the ET biosynthetic pathway [3,20]. Additionally, the expression of an ethylene receptor gene from pear, designated *PpERS*, was suppressed under 0.2 mM SA treatment [4]. However, whether SA regulates *EIN3* gene during fruit development remains unknown.

In higher plants, glucose has hormone-like activities and regulates many essential processes, such as senescence. Some studies have shown that there is an antagonistic interaction between glucose and ET. Furthermore, Yanagisawa (2003, [21]) reported that glucose enhances the degradation of EIN3, while ET enhances the stability of EIN3. However, whether glucose regulates *EIN3* gene during fruit development remains unknown. This study aims to elucidate the regulation of a pear *EIN3* gene (designated *PpEIN3b*) during fruit ripening and senescence via integrating SA, glucose, and ACC signaling, which would provide a candidate gene for the regulation of fruit ripening and senescence to obtain a long shelf life and improve the economic value.

## 2. Materials and Methods

### 2.1. Collection of Plant Materials

Pear (*Pyrus pyrifolia* Nakai. Whangkeumbae) fruit was collected at 30, 60, 90, 120, 130, 140, 145, and 150 days after full bloom from the experimental farm of the Agricultural University of Hebei, China. Fruit that grows 150 days after full bloom is naturally mature. After the natural harvest, the pear fruits were placed at room temperature for separate collection 5, 10, 15, 20, 25, and 30 days after harvest. Diseased fruit and controls were screened from the above 20 days after harvest. The mesocarp of pears was collected for further study. Young shoots, stems, and leaves, petals, and anthers were derived from the same pear trees of the local orchard. These samples were ground into powder with liquid nitrogen for RNA isolation.

### 2.2. Fruit Treatment

Mesocarp discs were collected from pear fruit at 150 days after full bloom with a hole punch and separated into two parts. One part was dipped into 0.002, 0.02, 0.2, and 2.0 mM SA (Biotopped) solution for 12 h for treatment. Control mesocarp discs were dipped into ddH_2_O for 12 h. The other part was treated with 0.2 mM SA for 3, 12, and 24 h. Untreated control discs were dipped into distilled water immediately.

Mesocarp discs were collected from pear fruit at 10 days after harvest with a hole punch and were subjected to 5, 10, 15, and 20% glucose solution for treatment for 12 h. Untreated discs were dipped into distilled water for 12 h as control.

Mesocarp discs were collected from pear fruit 145 days after full bloom with a hole punch and were treated with 0.1, 0.5, 1.0, 2.0, and 10.0 mM ACC (Sigma-Aldrich Corp. St. Louis, MO USA) for 12 h. Control mesocarp discs were dipped into distilled water for 12 h.

Mesocarp discs from 20 fruits of the same pear tree were collected with a hole punch and subjected to SA, ACC, and glucose treatment. Total RNA was extracted from the treated mesocarp discs and controls. Disc collection, treatment, and RNA extraction were repeated three times. The data were input into SPSS software 19.0 (IBM, Armonk, New York, USA), and *t* test of independent samples was performed for statistical inference.

### 2.3. Isolation of PpEIN3b cDNA

Total RNA was isolated from pear fruit 150 days after full bloom under 0.2 mM SA treatment. Poly(A)^+^ mRNA was purified from a pool of total RNA with an mRNA purification kit (Qiagen, Hilden, Germany). Complementary DNA (cDNA) was synthesized. About 1000 cDNA clones were screened from the pear fruit cDNA for sequencing (data not shown). One *EIN3* clone with a complete sequence was identified.

### 2.4. RNA Extraction and Quantitative RT-PCR Analysis

Total RNA was extracted from young shoots, stems, and leaves, petals, anthers, and developing mesocarp of pear by methods described previously [22].

Tissue special expression profiling of *PpEIN3b* in young shoots, stems, and leaves, petals, and anthers during different stages of fruit development was carried out by quantitative RT-PCR (qRT-PCR). SYBR-Green fluorescent intercalating dye (Toyobo Co. Ltd., Osaka, Japan) was used in the detection system (Mastercycler ep realplex 4, Eppendorf AG, Hamburg, Germany) for the experiment. *PpUBI* (GenBank accession number: AF195224) was used as a standard control in the RT-PCR; mRNA was reverse transcribed into cDNA that was used as a template in PCR with gene-specific primers (*PpEIN3b* P1: 5′-TTACCTCAGGTTAAGGGAGAGCTC-3′; P2: 5′-CCGGTATGGACAATTCAACTGATG-3′. *PpUBI* P1: 5′-TGCAGATCTTCGTGAAAACCCTAAC-3′; P2: 5′-CATAGCACTTGCGGCAGATCATCT-3′). RT-PCR was carried out using a described method [22].

### 2.5. DNA Sequencing and Protein Analysis

The sequences of isolated pear *EIN3* cDNA and its deduced protein were analyzed with DNAstar software 12.1 (DNAstar Inc., Madison, WI, USA). The conserved domain was performed with NCBI Conserved Domain Search (http: //blast.ncbi.nlm.nih.gov/Blast.cgi). Protein sequence homology analysis was performed with Clustal Omega (http://www.ebi.ac.uk/Tools/msa/clustalo/) and protein motif analysis was performed with motif scan (http://myhits.isb-sib.ch/cgi-bin/motif_scan). With the Clustal O (1.2.4) program, 22 EIN3 sequences from different plants were aligned, and then the neighbor-joining evolutionary relationships of the EIN3s were determined by Clustal Omega.

## 3. Results

### 3.1. Isolation and Characterization of PpEIN3b

**Figure 1:**
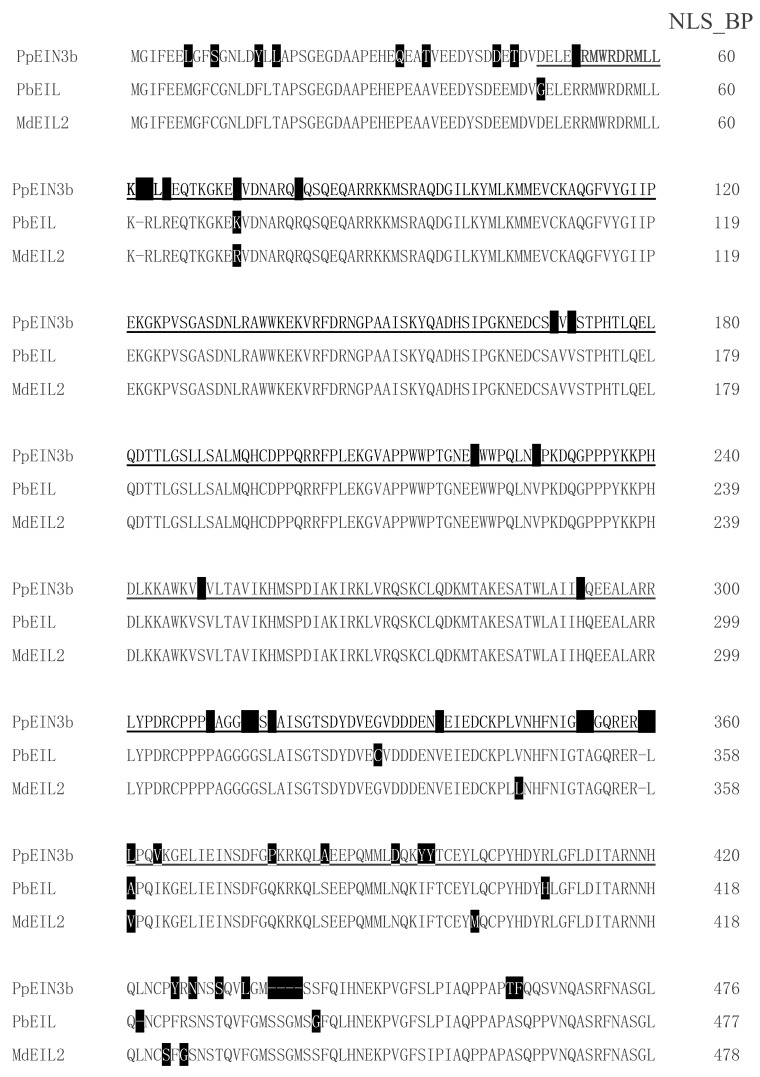

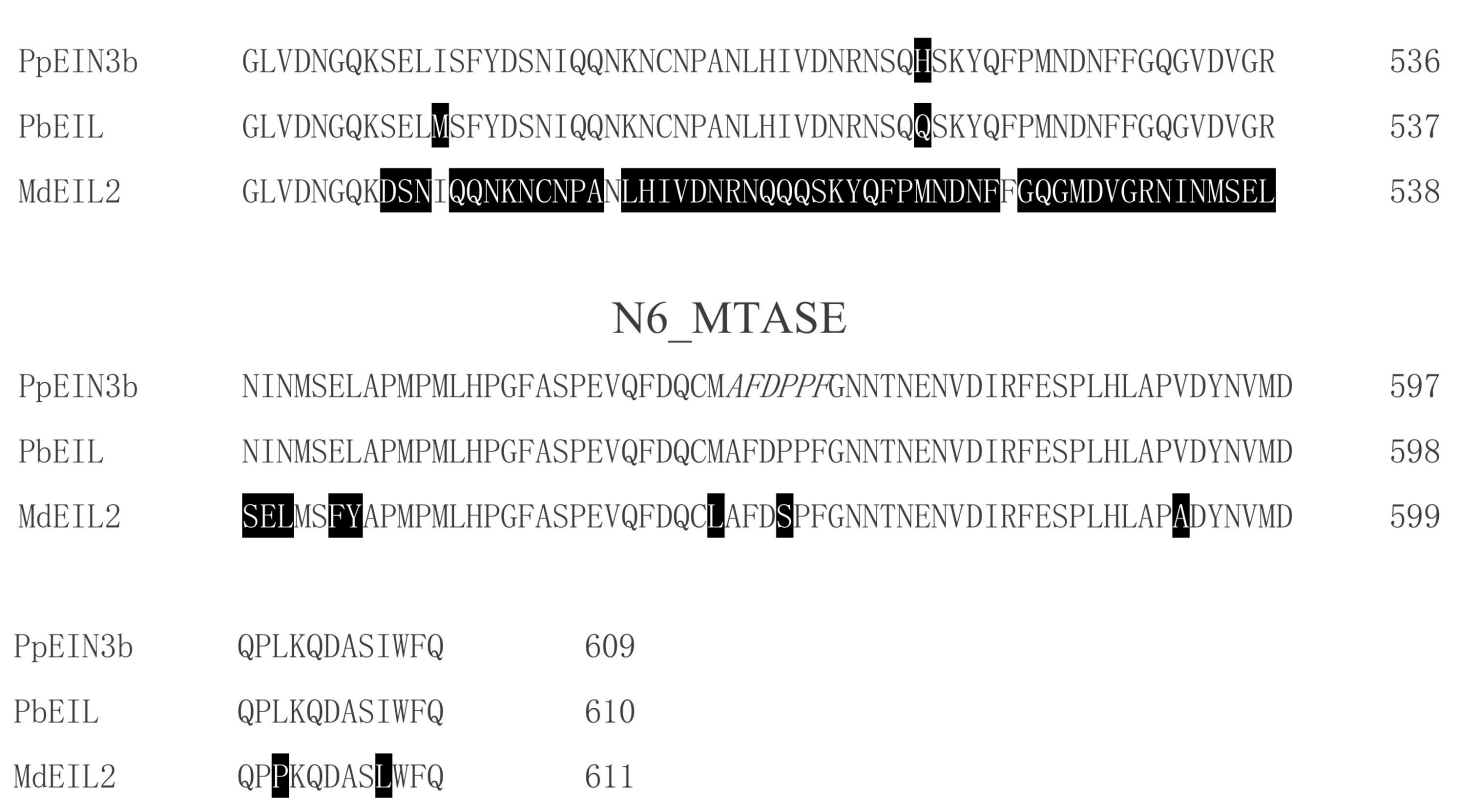
Sequence alignment of three fruit ethylene insensitive 3 (EIN3)/EIN3-like (EIL) proteins. Sequences of plant EIN3/EILs were aligned. Amino acid substitutions are highlighted in black. EIN3 domain is underlined. Bipartite nuclear localization signal profile (NLS_BP, K51RMWRDRMLLKKKLK65) is in bold and N-6 adenine-specific DNA methylase signature (N6_MTASE, M564AFDPPF570) is in italic. Accession numbers of plant EIN3 proteins in GenBank are: Sand pear PpEIN3b (KT726839, *Pyrus pyrifolia*), white pear PbEIL (XP_009339246, *Pyrus bretschneideri*), and apple MdEIL2 (GU732485, *Malus* × *domestica*).

By screening and sequencing the pear fruit cDNA pool, an *EIN3* cDNA was isolated and designated as *PpEIN3b* (GenBank accession number: KT726839). *PpEIN3b* cDNA encodes a protein with 609 amino acids. PpEIN3b shares relatively high homology (91.91% identity) with white pear EIL (XP_009339246, *Pyrus bretschneideri*) at the amino acid level. PpEIN3b also shares high homology (90.61% identity) with apple MdEIL2 (GU732485, *Malus* x *domestica*) at the amino acid level. As shown in Figure 1, PpEIN3b contains conserved EIN3 domain. Interestingly, PpEIN3b also contains a bipartite nuclear localization signal profile (NLS_BP, K_51_RMWRDRMLLKKKLK_65_) and an N-6 adenine-specific DNA methylase signature (N6_MTASE, M_564_AFDPPF_570_).

### 3.2. Phylogenetic Relationships of PpEIN3b with other Plant EIN3s

**Figure 2:**
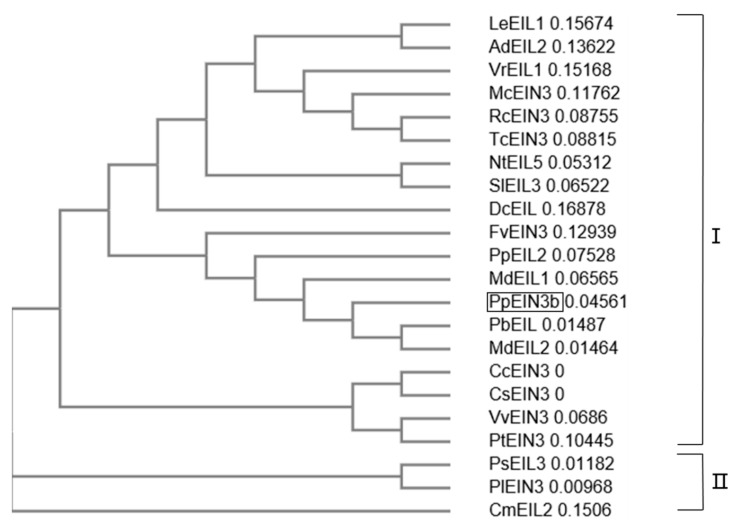
Phylogenetic relationships of PpEIN3b with other EIN3s/EILs. The neighbor-joining evolution tree was constructed in Clustal Omega and divided into two clades, I and II. GenBank accession numbers of plant EIN3s/EILs are: *PpEIN3b* (*Pyrus pyrifolia*, KT726839), LeEIL1 (FJ890314, *Lithospermum erythrorhizon*), AdEIL2 (EU887511, *Actinidia deliciosa*), VrEIL1 (AF467784, *Vigna radiate*), McEIN3 (KF595122, *Momordica charantia*), RcEIN3 (XM_002530146, *Ricinus communis*), TcEIN3 (XM_007016620, *Theobroma cacao*), NtEIL5 (AY248907, *Nicotiana tabacum*), SlEIL3 (NM_001247617, *Solanum lycopersicum*), DcEIL (AB525913, *Daucus carota*), FvEIN3 (XM_004306392, *Fragaria vesca*), PpEIL2 (EF031066, *Prunus persica*), MdEIL1 (KC128858, *Malus x domestica*), PbEIL (*Pyrus bretschneideri*, XP_009339246), MdEIL2 (GU732485), CcEIN3 (XM_006432473, *Citrus clementina*), CsEIN3 (XM_006471230, *Citrus sinensis*), VvEIN3 (XM_002276344, *Vitis vinifera*), PtEIN3 (XM_006379360, *Populus trichocarpa*), PsEIL3 (JQ771471, *Paeonia suffruticosa*), PlEIN3 (JX445144, *Paeonia lactiflora*), and CmEIL2 (AB063192, *Cucumis melo*).

An analysis of the phylogenetic relationships between PpEIN3b and other EIN3s previously reported in plants is shown in Figure 2. The first subgroup of the plant EIN3 tree comprises PpEIN3b, LeEIL1 (FJ890314, *Lithospermum erythrorhizon*), AdEIL2 (EU887511, *Actinidia deliciosa*), VrEIL1 (AF467784, *Vigna radiate*), McEIN3 (KF595122, *Momordica charantia*), RcEIN3 (XM_002530146, *Ricinus communis*), TcEIN3 (XM_007016620, *Theobroma cacao*), NtEIL5 (AY248907, *Nicotiana tabacum*), SlEIL3 (NM_001247617, *Solanum lycopersicum*), DcEIL (AB525913, *Daucus carota*), FvEIN3 (XM_004306392, *Fragaria vesca*), PpEIL2 (EF031066, *Prunus persica*), MdEIL1 (KC128858), PbEIL (XP_009339246, *Pyrus bretschneideri*), MdEIL2, CcEIN3 (XM_006432473, *Citrus clementina*), CsEIN3 (XM_006471230, *Citrus sinensis*), VvEIN3 (XM_002276344, *Vitis vinifera*), and PtEIN3 (XM_006379360, *Populus trichocarpa*). PsEIL3 (JQ771471, *Paeonia suffruticosa*), PlEIN3 (JX445144, *Paeonia lactiflora*), and CmEIL2 (AB063192, *Cucumis melo*) occupy another clade of the tree, which suggests that PpEIN3b might have diverged earlier from the other EIN3s during evolution.

### 3.3. PpEIN3b Expression is Regulated during Fruit Ripening and Senescence

**Figure 3:**
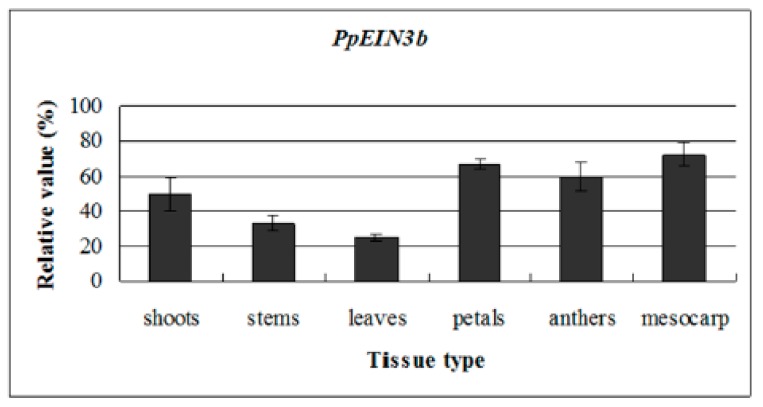
qRT-PCR analysis of expression of *PpEIN3b* in pear. Total RNA was extracted from shoots, young stems, young leaves, petals, anthers, and mesocarp. Relative value of expression of *PpEIN3b* in pear tissues is shown as a percentage of *PpUBI* expression. Mean values and standard deviation (SD) (bar) are shown from three independent experiments.

To reveal the *PpEIN3b* expression patterns in all tissues, qRT-PCR analysis was performed. The experimental results show that *PpEIN3b* is preferentially expressed in the mesocarp of pear fruit. Relatively high expression signals were detected in petals and anthers. *PpEIN3b* transcripts also accumulate in young shoots, stems, and leaves (Figure 3).

**Figure 4:**
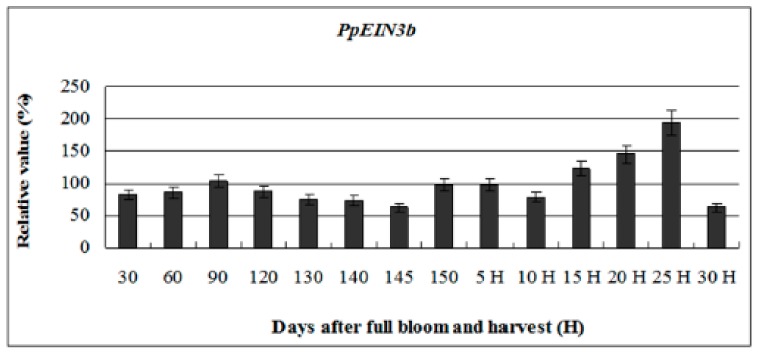
qRT-PCR analysis of expression of *PpEIN3b* during fruit development. Relative value of expression of *PpEIN3b* in pear fruit is shown as percentage of *PpUBI* expression. Mean values and SD (bar) are shown from three independent experiments.

To investigate whether the expression of *PpEIN3b* is regulated during fruit ripening and senescence, further analysis of its expression pattern during fruit development was performed. The experimental results (Figure 4) reveal that *PpEIN3b* has stationary expression activity during early fruit development (30–145 days after full bloom). However, with fruit ripening and senescence, the expression of the *PpEIN3b* gene increased to the highest level (relative value of 193.64) 25 days after harvest. With fruit softening, the expression of *PpEIN3b* gene sharply decreased to a relatively low level (relative value of 62.66). These results suggest that the *PpEIN3b* gene might play a crucial role in fruit ripening and senescence of pear.

### 3.4. Expression of PpEIN3b in Fruit is Inhibited by SA

**Figure 5:**
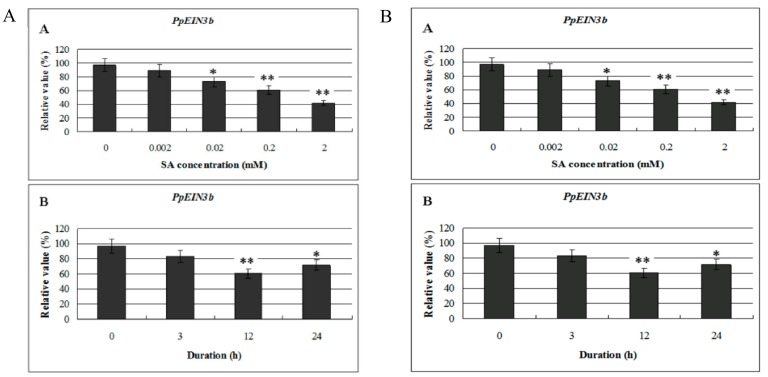
(**A**) qRT-PCR analysis of expression of *PpEIN3b* in fruit under SA treatment for 12 h. Relative values of expression of *PpEIN3b* in fruit 150 days after full bloom treated with 0, 0.002, 0.02, 0.2, and 2.0 mM SA for 12 h are shown as percentage of *PpUBI* expression. (**B**) qRT-PCR analysis of expression of *PpEIN3b* in fruit under 0.2 mM SA treatment. Relative values of expression of *PpEIN3b* in fruit 150 days after full bloom treated with 0.2 mM SA for 0, 3, 12, and 24 h are shown as percentage of *PpUBI* expression. Mean values and standard errors (bar) are shown from three independent experiments. Independent *t* tests for equality of means demonstrated significant (* *p* value < 0.05) or very significant (** *p* value < 0.01) difference between control and treated fruit. SA: salicylic acid.

To study whether the expression of isolated *PpEIN3b* is regulated by SA, mesocarp of fruit 150 days after full bloom was subjected to SA treatment. Total RNA was extracted from treated and nontreated mesocarp discs of pear, and then reverse transcribed into cDNA for qRT-PCR analysis. As shown in Figure 5A, the expression of *PpEIN3b* was inhibited significantly by 0.02, 0.2, and 2.0 mM SA for 12 h. Furthermore, *PpEIN3b* was inhibited by 0.2 mM SA for 12 and 24 h (Figure 5B), revealing that pear *EIN3* has different time course expression patterns in response to SA.

### 3.5. Expression of PpEIN3b in Fruit is Downregulated by Glucose

**Figure 6:**
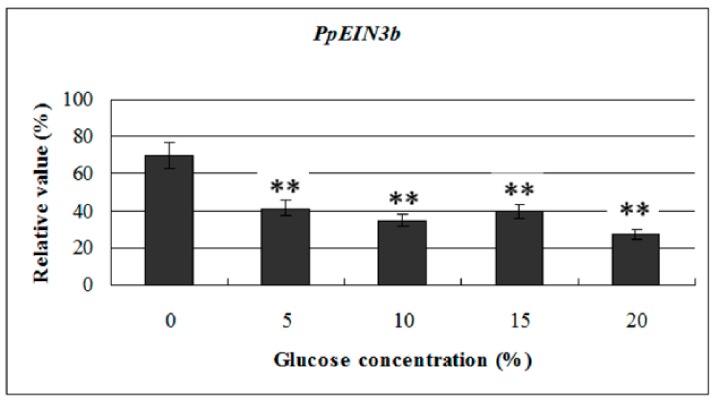
qRT-PCR analysis of expression of *PpEIN3b* in fruit under glucose treatment for 12 h. Relative values of expression of *PpEIN3b* in fruit 10 days after harvest treated with 0, 5%, 10%, 15%, and 20% glucose for 12 h shown as percentage of *PpUBI* expression. Mean values and standard errors (bar) are shown from three independent experiments. Independent *t* tests for equality of means show very significant difference (** *p* value < 0.01) between control and treated fruit.

To investigate the effects of glucose on *PpEIN3b* activity in pear fruit, we examined its expression profile in fruit 10 days after harvest treated with glucose. The experimental results show that the transcript levels of *PpEIN3b* were significantly downregulated by 5, 10, 15, and 20% glucose treatment for 12 h, and the lowest with 20% glucose (Figure 6). The results suggest that *PpEIN3b* might be involved in the response to glucose signaling during fruit ripening and senescence of pear.

### 3.6. Expression of PpEIN3b in Fruit is Upregulated by ACC

**Figure 7:**
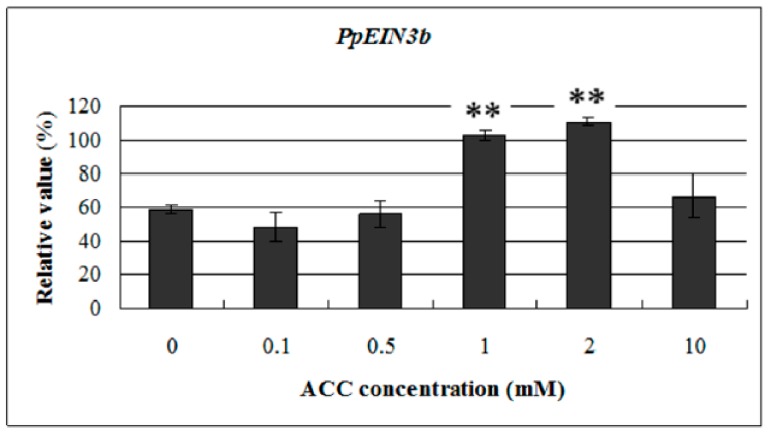
qRT-PCR analysis of *PpEIN3b* expression in fruit under ACC treatment. Relative values of *PpEIN3b* expression in fruit 145 days after full bloom treated for 12 h with 0.1, 0.5, 1.0, 2.0, and 10.0 mM ACC are shown as percentage of *PpUBI* expression. Mean values and standard errors (bar) are shown from three independent experiments. ** Very significant difference between treated and control fruit (*p* < 0.01) by *t* test. ACC: -aminocyclopropane-1-carboxylic acid

To study the response of *PpEIN3b* to ethylene at the transcriptional level, we examined its expression profile in pear fruit at 145 days after full bloom with treatment of 0.1, 0.5, 1.0, 2.0, and 10.0 mM ACC for 12 h. Total RNA was extracted from treated and nontreated mesocarp discs and then reverse transcribed into cDNA for qRT-PCR analysis. The results (Figure 7) indicate that *PpEIN3b* expression was upregulated at 12 h with 1.0 and 2.0 mM ACC but was not affected with 0.1, 0.5, and 10.0 mM ACC for 12 h. These results show that *PpEIN3b* expression was induced by some concentrations of ACC in pear fruit.

## 4. Discussion

The members of the EIN3 super-family are homologous and conservative. For example, PpEIN3b shares relatively high homology (91% identity) with white pear EIL1 protein (XP_009339246, *Pyrus bretschneideri*) at the amino acid level. DC-EIL1/2 (*Dianthus caryophyllus*, AY728191) shares 98% identity with DC-EIL1 (AF261654), 62% identity with DC-EIL3 (AY728192), and 60% identity with DC-EIL4 (AY728193). DC-EIL3 shares 100% identity with Dc106 (CF259543), 61% identity with DC-EIL1, and 59% identity with DC-EIL4. DC-EIL4 shares 60% identity with DC-EIL1 [23]. All EIN3 proteins contain conserved EIN3 domains.

Plant *EIN3*s and *EIL*s show different kinds of expression profiles. In this study, *PpEIN3b* displayed a tissue-preferential expression pattern and was preferentially expressed in fruit. Furthermore, *PpEIN3b* expression was developmentally regulated during fruit ripening and senescence. However, some fruit *EIN3* genes have shown constitutive expression during fruit development. Four *EIL*s were isolated from kiwi fruit (*Actinidia deliciosa*) and designated *AdEIL*s. *AdEIL*s were constitutively expressed during fruit development and ripening [11]. These results support a role for *EIN3*s and *EIL*s in the regulation of fruit development.

In plants, *EIN3* expression is regulated by phytohormones. *PpEIN3b* expression was downregulated by SA treatment in pear fruit, while the accumulation of *PpEIN3b* transcripts significantly increased under ACC treatment, suggesting that *PpEIN3b* might be a negative regulator in delaying fruit ripening and senescence to lengthen shelf life. The changing *PpEIN3b* expression by the treatment is crucial for regulating fruit ripening and senescence. The expression levels of *PpEIN3b* can be reduced by SA treatment to delay the ripening and senescence of pear fruit. SA treatment suppressed ACS and ACO activity in ethylene biosynthesis, retarding the climacteric increase in ethylene production and delaying ripening and senescence in kiwi fruit [24]. In banana (*Musa acuminata*) fruit, *MA-EIL2* is a ripening and ethylene-inducing gene, unique within the *EIL* gene family [25]. Tomato *LeEIL*s show some functional redundancy and can modulate ethylene response and fruit development [26]. *EIN3*s and *EIL*s encode key transcriptional factors of ethylene signaling, and homologues have been identified in many plant species. They function downstream of ethylene receptors [27]. The stability of EIN3 was enhanced by ethylene treatment [21]. *DC-EIL3* mRNA showed significant accumulation upon ethylene exposure, during flower development, and upon pollination in petals and styles [23].

**Figure 8:**
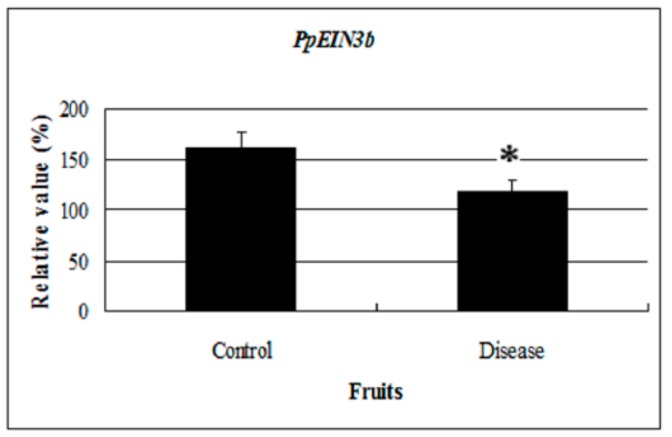
qRT-PCR analysis of *PpEIN3b* expression in diseased fruit. Relative values of *PpEIN3b* expression in diseased fruit 20 days after harvest are shown as percentage of *PpUBI* expression. Mean values and standard errors (bar) are shown from three independent experiments. Independent *t* tests for equality of means show significant difference (* *p* value < 0.05) between control and diseased fruit.

Interestingly, *EIN3* and *EIL* expression was regulated by sugar treatment in wounded, senescing, or diseased plant tissue. Stability of EIN3 was inhibited in the presence of glucose [21]. Flowers treated with sucrose showed a 2 days delay in the accumulation of *DC-EIL3* transcripts compared with control flowers. Decreasing levels of *DC-EIL3* mRNA were found in wounded leaves and ovaries of senescing flowers whenever ethylene levels increased [23]. Altogether, glucose treatment could reduce ethylene signaling, inhibiting senescence and extending the shelf life of fruit. In this study, *PpEIN3b* expression was repressed by glucose treatment in pear fruit and in diseased fruit (Figure 8). The expression of *PpEIN3b* was downregulated in diseased fruit, suggesting that the *PpEIN3b* gene might be involved in the response to disease signaling during ripening and senescence of pear fruit and its expression could be regulated to obtain disease-resistance and a long shelf life of pear fruits.

## 5. Conclusions

In this study, one gene encoding putative ETHYLENE INSENSITIVE3 (EIN3) protein was cloned from sand pear (*Pyrus pyrifolia*) and designated *PpEIN3b*. The deduced PpEIN3b contains a conserved EIN3 domain, a bipartite nuclear localization signal profile (NLS_BP), and N-6 adenine-specific DNA methylase signature (N6_MTASE) motifs. PpEIN3b belongs to the EIN3 super-family by phylogenetic analysis. qRT-PCR analysis revealed that *PpEIN3b* was preferentially expressed in fruit. Additionally, its expression was developmentally regulated during fruit ripening and senescence. Further study demonstrated that the transcript levels of *PpEIN3b* significantly increased under ACC treatment, while its expression was remarkably repressed by SA and glucose treatments in pear fruit and in diseased fruit. These data suggest that *PpEIN3b* might be involved in the response to ACC, SA, and glucose signaling during the development of pear fruit. In summary, this study reveals the expression and regulation profiles of *PpEIN3b* and provides a candidate gene for regulating fruit ripening and senescence to lengthen the shelf life and improve the economic value of fruit.
